# Robust Point Cloud Registration Network for Complex Conditions

**DOI:** 10.3390/s23249837

**Published:** 2023-12-15

**Authors:** Ruidong Hao, Zhongwei Wei, Xu He, Kaifeng Zhu, Jiawei He, Jun Wang, Muyu Li, Lei Zhang, Zhuang Lv, Xin Zhang, Qiwen Zhang

**Affiliations:** 1 Changchun Institute of Optics, Fine Mechanics and Physics, Chinese Academy of Sciences, Changchun 130033, Chinawj@ciomp.ac.cn (J.W.); zhangqiwen22@mails.ucas.ac.cn (Q.Z.); 2 University of Chinese Academy of Sciences, Beijing 100049, China; 3 College of Electronic and Information Engineering, Suzhou University of Science and Technology, Suzhou 215009, China; 4 Faculty of Electronic Information and Electrical Engineering, Dalian University of Technology, Dalian 116024, China; muyuli@dlut.edu.cn

**Keywords:** point cloud, point cloud registration, multi-scale feature extraction, coarse-to-fine registration

## Abstract

Point cloud registration is widely used in autonomous driving, SLAM, and 3D reconstruction, and it aims to align point clouds from different viewpoints or poses under the same coordinate system. However, point cloud registration is challenging in complex situations, such as a large initial pose difference, high noise, or incomplete overlap, which will cause point cloud registration failure or mismatching. To address the shortcomings of the existing registration algorithms, this paper designed a new coarse-to-fine registration two-stage point cloud registration network, CCRNet, which utilizes an end-to-end form to perform the registration task for point clouds. The multi-scale feature extraction module, coarse registration prediction module, and fine registration prediction module designed in this paper can robustly and accurately register two point clouds without iterations. CCRNet can link the feature information between two point clouds and solve the problems of high noise and incomplete overlap by using a soft correspondence matrix. In the standard dataset ModelNet40, in cases of large initial pose difference, high noise, and incomplete overlap, the accuracy of our method, compared with the second-best popular registration algorithm, was improved by 7.0%, 7.8%, and 22.7% on the MAE, respectively. Experiments showed that our CCRNet method has advantages in registration results in a variety of complex conditions.

## 1. Introduction

Rigid registration of point clouds is an important aspect in the field of 3D reconstruction and computer vision [1,2], aligning multiple point clouds with different viewpoints or different poses under the same coordinate system [3]. With the development of 3D acquisition technology in recent years, it has become very convenient to acquire point cloud data. Point cloud data is characterized by a simple format and good versatility and it is used in a large number of applications in 3D information storage, display, and processing. However, point clouds collected by remote sensing equipment usually have a single view. There are often differences in position and attitude between point clouds acquired from different viewpoints. If two or more point clouds need to be stitched together to form a complete point cloud, point cloud registration technology enables the stitching and integration of this data. 

Early applications of point cloud registration were related to information modeling in the construction industry, providing assistance in the mining industry, etc., but with the development of 3D reconstruction, robot navigation, autonomous driving technology, and virtual reality technology [4,5,6], point cloud registration algorithms are currently more widely used. Robust, reliable, and accurate point cloud registration algorithms can generate more complete 3D scene models, build accurate positioning maps, and contribute to path planning. However, for constructing point cloud models of an object, the existing point cloud registration algorithms do not work well in complex situations where the initial positions differ too much, contain high noise, or incompletely overlap. Therefore, it is of great significance to study point cloud registration algorithms for complex conditions.

Point clouds make registration a challenge due to their unorganized and unstructured data form. Traditional point cloud registration algorithms still have some limitations in terms of registration accuracy in complex situations. Benefiting from the development of deep learning, there has been a huge advancement in various intelligent processing techniques for point clouds. The emergence of PointNet [7] has led to the rapid development of intelligent point cloud learning, and PointNet and its deformations have been applied to point cloud registration algorithms. For example, the pioneering work PointNetLK [8], PCRNet [9], DCP [10] and other algorithms have been successfully applied to point cloud registration, and have the advantage of accuracy and speed compared with traditional algorithms such as ICP. Later, a series of algorithms developed on this basis further improved the various indexes of registration, but the registration problem in complex situations needs further research. Especially in cases of a too-large difference in the initial position, obvious noise, or incomplete overlap, these algorithms suffer from the problems of poor accuracy, falling into the local optimum, or even cannot be registered and mismatched. Therefore, it is still an extremely challenging task to utilize point cloud deep learning algorithms to deal with the point cloud registration problem in complex situations.

In this paper, in order to solve the above problems, we designed a new point cloud registration algorithm based on deep learning, which matches two pairs of point clouds from coarse to fine in an end-to-end form. Different from the existing point cloud registration methods, our algorithm pays more attention to the registration under the conditions of a large initial position difference, high noise, and low overlap. For this reason, we designed a feature extraction module, a coarse registration module, and a fine registration module suitable for a point cloud registration algorithm. In order to extract more features for registration, we calculated the local information in the multi-scale neighborhood to extract more local features. Most of the point cloud registration algorithms need accurate point correspondence, which is a very time-consuming process. The soft correspondence connection module designed by us can avoid this problem. In addition, for the registration of high noise and incomplete overlaps, the soft correspondence connection module can effectively avoid the one-to-one correspondence and can better deal with the registration work. In the coarse registration decoding module, the quaternion of rotation is predicted by MLPs, a pair of point clouds are rotated first, and then the final rotation matrix is predicted by SVD in fine registration. We have carried out a large number of experiments on ModelNet40 and verified the effectiveness of each module under the same conditions. The results of the experiments show that our point cloud registration network is able to perform accurate registration in different complex situations, and its accuracy is better than the existing popular algorithms. The contributions of this paper can be summarized as follows:

1. A new point cloud registration network, CCRNet, is proposed, which registers two point clouds in a coarse-to-fine registration manner. Compared with previous methods, our method can register point clouds robustly and accurately in complex situations without iteration.
2. A multi-scale feature extraction module is proposed, which combines the point cloud neighborhood map and the Transformer structure to obtain the features of different scales of the point cloud and the information of the relationship between two point clouds, which can greatly improve the ability of point cloud registration.
3. The soft correspondence connection in the fine registration module combines the CBAM structure suitable for point clouds, which improves the accuracy of the point correspondence in the soft registration module, and also improves its ability in incomplete overlapping point cloud registration.
4. The experimental results on the general dataset show that our network, CCRNet, is superior to the current popular algorithms in point cloud registration, and show its effectiveness.

### Related Work

**Classical registration methods.** Among the traditional registration methods, ICP (P2P-ICP) [11] is the most commonly used classical method. It estimates the transformation matrix by minimizing the sum of squares of the Euclidean distances between corresponding points and then obtains the final transformation matrix by means of multiple iterations and optimization. However, the results of the ICP algorithm do not perform well for point clouds containing noise, outliers, or initial point cloud poses that are far apart. In order to further solve these problems, researchers have proposed many variants of ICP algorithms, such as ICP (point to plane) [12] and GTLS-ICP [13], which are able to improve the registration accuracy, and both algorithms have improved robustness and the ability to suppress noise. The RPM algorithm [14] proposed by GOLD avoids falling into local minima during iteration via the allocation of reinforcement points. In order to improve the efficiency of point cloud registration, many researchers have improved the speed of point cloud registration by introducing KD-tree and Anderson algorithms, such as AA-ICP [15]. A more representative algorithm in the ICP variant is Go-ICP [16], which utilizes an octree data structure, among other things, to solve the problem of local minima, but it is difficult to solve the problem of widely spaced initial poses. Most of the many variants of the ICP algorithms rely on the initial pose, while the ICP-based algorithms cannot correctly compute the point correspondence for two point clouds that do not overlap exactly, thus limiting the accuracy of the registration results.

4PCS [17] is a classical coarse registration algorithm based on the RANSAC algorithm, which has the ability to be insensitive to noise and still be very robust to point clouds with large differences in initial attitude, but its algorithmic accuracy is associated with poorer and more time-consuming computations. NDT [18] is a discrete point cloud registration approach utilizing statistical probability. However, since the algorithm is divided in voxels, its optimization convergence is poor. Unlike ICP, NDT does not need to explicitly compute the correspondence point relationship, but it needs a good initial positional attitude, otherwise it can easily fall into local minima. With a classical algorithm for two point clouds with large differences in initial position and incomplete overlap, the registration effect will be greatly reduced, or even fail.

**Feature-based registration methods.** This class of registration methods usually includes feature detection, feature description, and feature matching. Feature-based registration methods are broadly categorized into those based on point features [19,20], line features [21,22], surface features [23,24], and texture features [25]. The difficulty of the feature-based registration approach is to extract the correct feature descriptors, and when the point clouds do not overlap completely, the extracted features are difficult to fix in the overlapping regions of the point clouds, resulting in feature matching failure. The feature-based registration approach is sensitive to noise, has high time complexity, and the quality of the extracted high-dimensional features is not as good as the emerging learning-based approach for extracting features in recent years.

**Learning-based registration methods.** With the boom in deep learning, researchers have conducted work [7,26] related to various intelligent processing of point clouds. Learning-based point cloud registration work has emerged. Learning-based point cloud registration methods can extract more high dimensional features of the point cloud compared to non-learning methods. Many early researchers replaced a portion of the traditional registration structure with a neural network, whereby learning-based registration methods can be categorized into partial learning and end-to-end learning.

**Partial learning.** The speed and computational power of the original method can be improved by replacing one part of the traditional point cloud registration method with a neural network. SE-GICP [27] combined PointNet with ICP algorithms to achieve good registration results. 3DMatch [28] employs a voxel-based keypoint description method to establish correspondence point relationships. 3DFeatNet [29] uses the method of key point extraction and description based on the original point cloud and takes a triad to train the network, which improves the registration ability to some extent. Meanwhile, some point cloud registration algorithms such as USIP [30], KPSNET [31], PPFNET [32], and PPF-FOLDNET [33] have sprung up.

**End-to-end learning.** Currently, end-to-end deep learning methods for point cloud registration are becoming popular, and most researchers are committed to embedding differentiable poses and positions estimation into the end-to-end learning pipeline rather than combining it with traditional point cloud registration methods. PointNetLK [8], based on the PointNet and LK [34] algorithms, performs the Jacobi computation only once and then iterates several times to find the estimated poses and positions, which greatly improves the registration efficiency. PCRNet [9] uses a PointNet encoder with an MLP decoder to predict the position and attitude, which is simple but still outperforms the ICP algorithm after a finite number of iterations. The DeepVCP [35] algorithm utilizes PointNet++ [36] to extract features and select better corresponding points by extracting more local features. DCP [10] borrows the idea of ICP, extracts features by DGCNN [37], adds the Transformer module to take contextual information, and finally, utilizes SVD decomposition to obtain the transformation matrix. RPM-Net [38] uses a deterministic degenerate scheduling allocation scheme, where the acquired features and parameters are used to compute the matching matrix and then estimate the correspondences. However, in the case where the initial point clouds are too far apart, contain noise, or are two incompletely overlapping point clouds, the matching cannot be performed well in most of the existing registration methods, and mismatching and matching failures can occur.

## 2. Methods

Given two point cloud pairs $P_{src}=\{x_{1},\ldots ,x_{n}\}\in R^{3}$ and $P_{\mathrm{tgt}}=\{y_{1},\ldots ,y_{n}\}\in R^{3}$, we wish to align the source point cloud to the target point cloud in the same coordinate system by rigid rotation (R) and translation (T). Ideally, the points in the point cloud pairs correspond to each other and can be expressed as the following equation:

(1) $$y_{i}=Rx_{i}+T+N_{i},$$

where $N_{i}$ is the noise vector.

Our goal is to compute the rotational translation matrices (R and T) among them to minimize the error on the least squares:

(2) $$error(R,T)=\frac{1}{N}\sum_{i=0}^{N}{\left\|Rx_{i}+T-y_{i}\right\|}^{2},$$

where N is the number of point clouds and $y_{i}$ is the point in $P_{\mathrm{tgt}}$.

### 2.1. Overview

**Figure 1:**
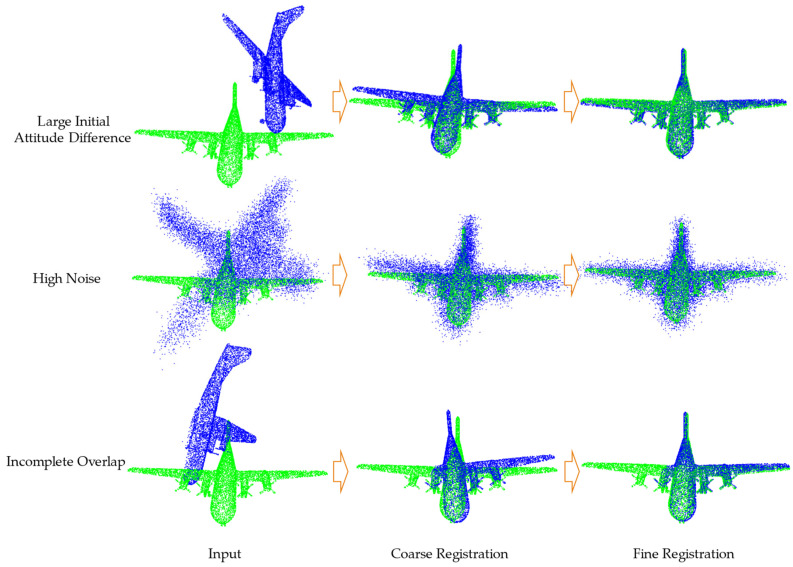
Two-step coarse-to-fine registration illustration.

The proposed end-to-end point cloud registration algorithm CCRNet is divided into two parts: coarse and fine registration. The network inputs two point clouds with different poses and positions, and by means of an encoder–decoder network structure, the rotation and translation matrices of the point clouds for coarse and fine registration are predicted respectively, and finally, the two point clouds are aligned to a uniform coordinate system. An illustration is shown in Figure 1 below.

**Figure 2:**
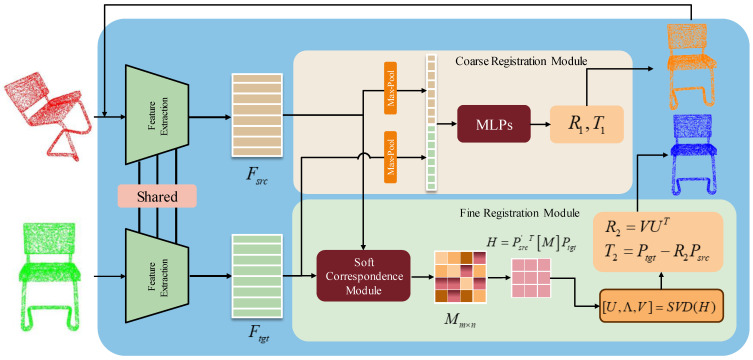
Overall framework structure of CCRNet, which is composed of a point cloud feature extraction module, a quaternion coarse registration module, and a SVD fine registration module. $F_{src}$ and $F_{tgt}$ denote the extracted point cloud feature maps, respectively. M is the soft corresponding probability matrix. H is the matrix to be decomposed by SVD.

The point cloud registration network is divided into three modules: a point cloud feature extraction module, a coarse registration module, and a fine registration module. The details of each module and the experimental details of the design are described in Figure 2 below.

### 2.2. Point Cloud Feature Extraction Module

**Figure 3:**
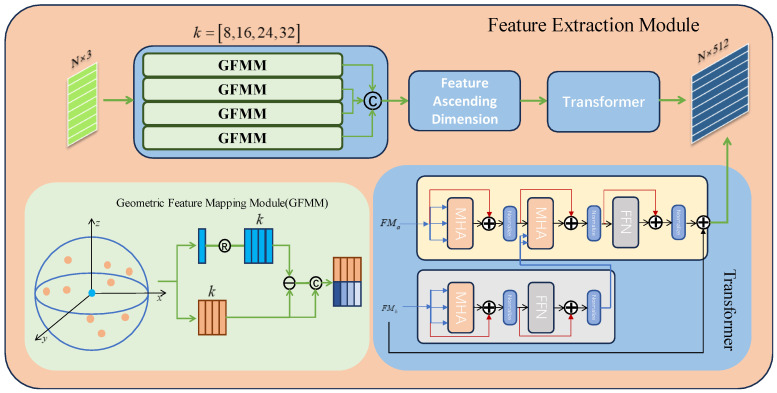
Multi-scale feature extraction module that contains GFMM and Transformer structures. k is the number of neighbors to find each point. © denotes the concatenation operation. ⊕ denotes the add operation. MHA denotes the Multi-Head Attention structures. FFN denotes the Location Based Feedforward Network.

In order to obtain richer and more accurate global and local features of the point cloud, the point cloud feature extraction module adopts the multi-scale extraction of local features to encode the two point clouds. Unlike the previous method of extracting features by PointNet++, the point cloud registration algorithm is not suitable for downsampling when extracting features, which will result in final features in which each point and feature are difficult to correspond to each other. The purpose of the point cloud registration algorithm feature extraction is not to extract the overall features of the point cloud, but to find the features of each point and each small region in the point cloud. Thus in this paper, we proposed a multi-scale point cloud feature extraction module in which multiple scales of KNN-graph structures are used to extract features as shown in the following Figure 3.

The neighborhood graph consists of a centroid coordinate $x_{i}$ and directed edges about it, and it maps low-dimensional features to higher dimensions via a function structure similar to that of a non-pooling PointNet. The formula is shown below:

(3) $$F_{x}=\phi (max\{\theta (x_{i},x_{j}-x_{i})|i=1,2,\ldots N.j=1,2,\ldots ,k\}),$$

where $x_{i}^{F}$ is the high-dimensional feature of a point in the point cloud, $x_{i}$ is a point in the point cloud, $x_{j}$ is the point $x_{i}$ proximity, max(⋅) is the maximum pooling operation, ϕ is an operation that maps low-dimensional features to high-dimensional features, and k is the number of proximity points to select, set to {8,16,24,32}. Then, the features at different scales are concatenated to finally extract a high-dimensional feature map F containing global features and local features at different scales. Since point cloud registration requires a source and a target point cloud, this module has two shared pipelines.

In order to improve the correspondence between the two point cloud high-dimensional features as shown in the Figure 3 above, we inserted the Transformer structure [39]. This module can utilize the multi-head attention mechanism to link the feature information between two point clouds, which allows the features of a single point cloud to communicate with each other and can improve the connection between the contexts of the two point clouds.

### 2.3. Coarse Registration Module

Our proposed point cloud registration is divided into two stages: coarse and fine registration. Coarse registration aims to roughly align two pieces of the point cloud at any initial position so that they are roughly aligned, thus providing a good initial position for fine registration so as to obtain more accurate point-correspondence results.

As shown in Figure 2, in order to implement the end-to-end registration prediction algorithm, we predicted the coarsely aligned rotational quaternions q($q\in R^{4}$) and translation vectors t($t\in R^{3}$) via MLPs. The two feature maps $F_{src}$, $F_{tgt}$ are extracted by the point cloud feature extraction module, and the global feature vectors $v_{src}$, $v_{tgt}$ of the two point clouds are obtained by maximum pooling (max-pooling). We concatenate the eigenvectors of the two point clouds and then predict the rotation quaternion q and translation vector t by MLPs. The formulas are as follows:

(4) $$q,t=MLPs(concat[\max(F_{src}),\max(F_{tgt})]),$$

where max(⋅) is the maximum pooling operation. The predicted quaternions q and translation vectors t are then transformed into the rotational translation matrices $R_{1}$ and $T_{1}$, then recorded. After the point cloud $P_{src}$ is subjected to coarse registration rotation according to the predicted $R_{1}$ and $T_{1}$, the resulting $P_{src}^{\prime }$ is then subjected to fine registration prediction with $P_{tgt}$.

### 2.4. Fine Registration Module

The Fine Registration Module is designed to improve the accuracy of the registration by further aligning it on the basis of the coarse registration. In this paper, we designed a soft point correspondence module that predicts the correspondence of points in two point clouds by means of a point correspondence probability matrix, thus simplifying the computation of point correspondences similar to multiple iterations in ICP and some popular algorithms. The specific process is shown in Figure 1 with the following equation:

(5) $$M=CBAM(Softmax(P_{src}^{\prime }{}^{T}P_{tgt}))$$

**Figure 4:**
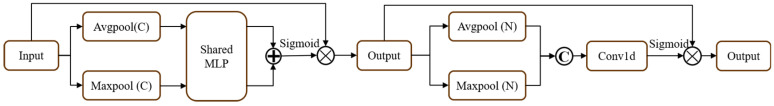
Schematic Structure of CBAM. © denotes the concatenation operation. ⊕ denotes the add operation. ⊗ denotes the multiply operation.

The soft point correspondence module consists of the point correspondence matrix M of $P_{src}^{\prime }$ and $P_{tgt}$. Each row in the M matrix represents the probability vector corresponding a point in $P_{src}^{\prime }$ to a point in $P_{tgt}$. The most probable correspondences of the point pairs can be predicted by training the correspondence matrix M as a way to find the correspondence with the highest probability. In this paper, we also hope that the corresponding matrix M can be robust against noise and satisfy the effective registration in a case of incomplete overlap. Therefore, a CBAM attention module [40,41] applicable to point clouds was added to enhance the relationship between high probability point correspondences and weaken the weights of some non-overlapping point pairs as a solution to satisfy the registration operation under complex conditions. The CBAM structure applicable to point clouds is shown in Figure 4.

After we obtain the soft point correspondence matrix, we can use the SVD method to find the optimal rotation and translation matrices, where the matrix H is constructed using the following equation:

(6) $$H=X_{src}^{\prime }{}^{T}\left[M\right]X_{tgt},$$

where $X_{src}^{\prime }$ and $X_{tgt}$ are the point cloud matrices formed by centering the point sets $P_{src}^{\prime }$ and $P_{tgt}$, of size m×3 and n×3, and m and n are the number of points in the model. The SVD decomposition of H is next performed with the following equation:

(7) $$H=U\Lambda V^{T}$$

Based on the matrices U and V, the rotation matrix can be calculated as:

(8) $$R_{2}=VU^{T}$$

Simply verify $\det(R_{2})=1$ again. Finally the translation of the point cloud is calculated as:

(9) $$T_{2}=P_{tgt}-R_{2}P_{src}^{\prime }$$

We multiply the rotation matrices predicted by the coarse and fine registration twice to obtain the final rotation matrices R and T.

### 2.5. Training Loss

In this paper, the Earth Movers Distance (EMD) [42] was used as the loss function for the coarse registration stage. EMD is sensitive to the integrity of the point cloud shape, which helps improve the overlapping ratio between two point clouds $P_{\mathrm{src}}$ and $P_{\mathrm{tgt}}$ in the coarse registration stage. The EMD distance is defined as:

(10) $${ℒ}_{EMD}(P_{\mathrm{src}},P_{\mathrm{tgt}})=\underset{\phi :P_{\mathrm{src}}\to P_{\mathrm{tgt}}}{\min}\frac{1}{\left|P_{src}\right|}\sum_{x\in P_{src}}\left\|x-\phi (x)\right\|$$

In the fine registration stage, we use the error of the rotation matrix and the translation vector as the loss function, and we define the loss of the second stage as:

(11) $${ℒ}_{2}(R_{1},R_{2},t_{1},t_{2})={\left\|{\left(R_{2}R_{1}\right)}^{T}R_{g}-I\right\|}^{2}+{\left\|R_{2}t_{1}+t_{2}-t_{g}\right\|}^{2},$$

where I denotes the unit matrix. $R_{g}$ and $t_{g}$ are the ground truth of rotation and translation, respectively. $R_{1}$ and $t_{1}$ are predicted by the coarse registration and $R_{2}$ and $t_{2}$ are predicted by the fine registration.

So we define the total loss as:

(12) $${ℒ}_{total}={ℒ}_{EMD}(P_{\mathrm{src}},P_{\mathrm{tgt}})+{ℒ}_{2}(R_{1},R_{2},t_{1},t_{2})$$

## 3. Experiments

In this section, we demonstrate the robustness and effectiveness of our algorithms for clean data, noisy data, and incomplete overlaps by performing analysis and experiments on the standard dataset ModelNet40 [43] for point cloud registration in different environments.

### 3.1. Dataset

ModelNet40 [43] is the most commonly used point cloud dataset. It is one of the ModelNet family of datasets, which contains 40 different object categories with about 1000 3D models in each category of common furniture, electronics, animals, and so on. Each point cloud contains xyz coordinate information and gives the category label of each model for training and evaluation.

### 3.2. Metrics

In order to analyze and compare our results with the previous algorithms on the same scale, we selected six commonly used evaluation criteria for point cloud registration algorithms, namely, the mean square error (*MSE*), root mean square error (*RMSE*), and mean absolute error (*MAE*) for rotated Euler angles and translational vectors.

We converted the predicted rotation matrix R to the Euler angle $Eu_{pre}$. The ground truth is $Eu_{gt}$ and the rotation errors were calculated as follows:

(13) $$MSE\left(R\right)=\frac{1}{n}\sum_{i=1}^{n}{\left(Eu_{pre}-Eu_{gt}\right)}^{2},$$

(14) $$RMSE\left(R\right)=\sqrt{\frac{1}{n}\sum_{i=1}^{n}{\left(Eu_{pre}-Eu_{gt}\right)}^{2}},$$

(15) $$MAE\left(R\right)=\frac{1}{n}\sum_{i=1}^{n}|Eu_{pre}-Eu_{gt}|$$

$T_{pre}$ is the predicted translation vector and $T_{gt}$ is the ground truth of translation vector, so the translation error is as follows:

(16) $$MSE\left(t\right)=\frac{1}{n}\sum_{i=1}^{n}{\left(t_{pre}-t_{gt}\right)}^{2},$$

(17) $$RMSE\left(t\right)=\sqrt{\frac{1}{n}\sum_{i=1}^{n}{\left(t_{pre}-t_{gt}\right)}^{2}},$$

(18) $$MAE\left(t\right)=\frac{1}{n}\sum_{i=1}^{n}|t_{pre}-t_{gt}|$$

### 3.3. Implementation Details

We have analyzed the data processing of many point cloud registration algorithms, and most of the algorithms have the target point cloud $P_{\mathrm{tgt}}$ and the source point cloud $P_{\mathrm{src}}$ as completely overlapping point clouds, which are just retrained by a predetermined rotational translation. In this paper, it is argued that in reality the point cloud data acquired by the sensors are not exactly overlapping, and that there should be a certain amount of error or a different distribution between each point. So, we randomly sampled each point cloud model two different times, and then rotated and translated one of the sampled point cloud models, thus obtaining two point cloud datasets for input training. The rotation angle of the point cloud data is set to be randomized between [−45°, 45°] and the translation vector is set to be randomized between [−0.5, 0.5].

In order to ensure the fairness of the experiment, we had to re-train and re-evaluate the other reference algorithms according to the treatment of point cloud data as described above. Meanwhile, all algorithms only utilize the xyz data of the point cloud and do not utilize normal data. The iteration numbers of PointNetLK and PCRNet are set to 8. The DCP algorithm is divided into training v1 and v2.

Our framework is implemented in PyTorch framework and trained on NVDIA 3080Ti GPU. The Adam optimizer is used with an initial learning rate of 0.001, milestone set to [100, 200], and gamma of 0.1. In total, 300 epochs were trained.

### 3.4. Experimental Results

#### 3.4.1. Results on Clean Data of a Large Initial Attitude Difference

On the dataset ModelNet40 [43], our results were compared with other popular advanced point cloud registration algorithms where the initial rotation was set to a relatively large interval of [−45°, 45°], and the quantitative comparison results on clean data are shown in Table 1.

We compared our method with some traditional algorithms and popular deep learning algorithms in point cloud registration (all of which do not use normal information). This experiment proves that our algorithm is comprehensively ahead of other ICP-based and learning-based algorithms The Euler angle rotation *MSE(R)* is an order of magnitude ahead of the commonly used algorithm ICP and 7% ahead of the second-ranked DCPv2 method. The translation vector error *MSE(t)* is even farther ahead of the ICP-based algorithm and 8% ahead of the DCPv2. This is attributed to the more accurate features extracted by our extraction module and the structure of the two-stage registration, which plays a great role in the process of registration. In Table 1, we can see that the error of t in ICP is much higher than that of one of the other algorithms. This is because the ICP algorithm has a lot of registration failures, and the error after a failure is extremely large, which can inflate its average results.

As can be seen from Table 1, the accuracy of the FDCP algorithm based on feature descriptors is far ahead of the other algorithms, but its disadvantage is that its running speed is very slow. During the experiment, we found that the FDCP registration time of different point cloud models was different; some completed the registration within 2 s, while some point clouds took 350 s or longer to successfully register, which shows that this algorithm based on feature descriptors generally cannot meet the needs of real-time performance, which is why most registration algorithms based on deep learning are not compared with them.

#### 3.4.2. Results on Noise Data

In order to verify the robustness of various algorithms to noise, we added Gaussian noise with a mean value 0 and standard deviation [0, 0.02] to each point in the point cloud data, and the other conditions were consistent with the experimental conditions given above. We performed a comparison experiment, and the comparison data can be visualized in the following Table 2.

In the point cloud data after adding Gaussian noise, our method is still able to robustly register the point cloud. Compared to other point cloud registration algorithms, our method is able to continue to work in high noise conditions. And both the rotation error and translation error are smaller than other mainstream methods, which are 19% and 2.86% better than the second-best DCPv2 algorithm on the *MSE*, respectively. These experiments show that our method is very robust under noise and can perform point cloud registration stably and accurately.

In order to test the robustness and effectiveness of our algorithm under higher noise, we made a detailed comparison with the ICP algorithm and FDCP algorithm based on descriptors. We add weak to strong Gaussian noise with an absolute value within 0.5 and sigma range [0.01, 0.1] respectively.

In Table 3, we know that ICP and our algorithm are not affected by noise, running very fast, and having certain anti-interference ability. At the same time, our algorithm based on deep learning can more effectively adapt to the registration work under high noise. It is difficult for feature descriptor-based algorithms to register fast and accurately in cases of high noise because it is difficult for such algorithms to obtain usable descriptors, which is also verified by the FDCP algorithm. However, the feature level extracted by the registration algorithm based on deep learning is deeper and can solve this problem. In terms of running time, both our algorithm and the ICP algorithm are within 0.05 s, which can meet the real-time requirements and is far ahead of FDCP by hundreds of seconds. Therefore, compared with feature-based registration algorithm, we have advantages in high noise conditions and running speed.

#### 3.4.3. Results on Incomplete Overlapping Data

In practice, point cloud data often do not overlap completely, and we have taken this complication into account by inserting a random plane into a pair of point clouds, which randomly splits off 20% of the point cloud, so that we can obtain pairs of point clouds with an overlap of 80%. We conducted the same quantitative experiment as above and the results are shown in Table 4.

Incompletely overlapping point cloud registration is a great challenge for all algorithms, but our method still outperforms the others. This is because our correspondence matrix takes into account the problem of matching point cloud pairs in non-overlapping regions by giving higher probability assignment values to point cloud pairs with greater confidence, so that the computed H-matrix is more suitable for incompletely overlapping point cloud models. In terms of rotational error, our results are 21% lower than ICP on *MSE.* The large translation error of the ICP algorithm indicates its many matching failures, whereas our algorithm is able to guarantee the correctness of the translation based on the correct rotation. The experimental results prove that our algorithm is able to handle the incomplete overlapping point cloud registration problem and outperforms some popular registration algorithms.

## 4. Discussion

In order to further analyze and validate the effectiveness and intrinsic connectedness of our algorithm, we performed an experimental analysis of the registration with different initial angles and translations in the clean ModelNet40 dataset.

### 4.1. The Effect of the Initial Rotation Angle on the Results

**Figure 5:**
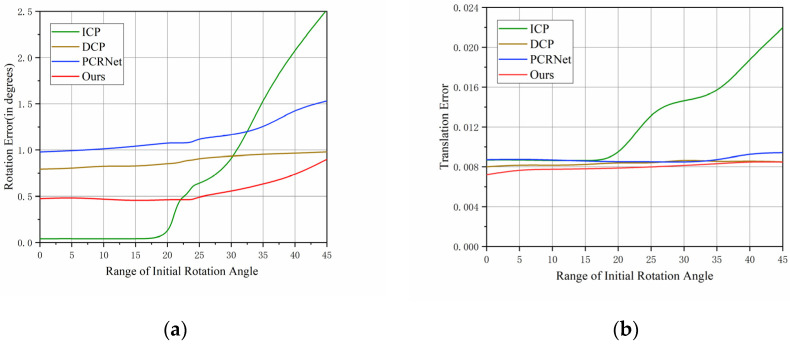
(**a**) The effect of the initial rotation angle on the final rotation error; (**b**) The effect of the initial rotation angle on the final translation error.

This experiment fixed the initial translation vector, which was set to [−0.5, 0.5], and it explores the relationship between the registration results and the initial rotation angle by changing the initial rotation angle sequentially from [−5°, 5°] to [−45°, 45°]. The experimental results are shown in Figure 5 below:

From Figure 5, we find that the results of the ICP algorithm are better than the other algorithms in the initial rotation angle range of [0, 20°], but after the initial rotation angle reaches 20 degrees or more, the rotation error and translation error of the ICP algorithm increase dramatically. This is because the ICP algorithm does not work well for large initial rotation angles, and there will be registration failures, which shows that the ICP is not stable enough for registration in some complex situations. Our algorithm is more stable and robust than the ICP algorithm, PCRNet, and DCP. It can still provide stable and accurate registration with large initial rotation angles, and the rotation error and translation error are smaller than those of the other methods.

### 4.2. The Effect of the Initial Rotation Angle on the Results

**Figure 6:**
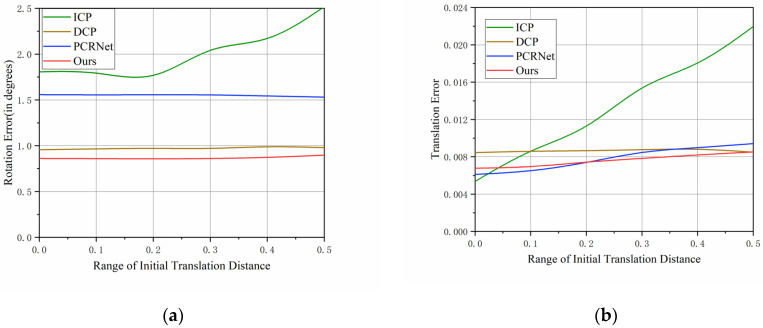
(**a**) The effect of the initial translation distance on the final rotation error; (**b**) The effect of the initial translation distance e on the final translation error.

This experiment fixed the initial rotation angle, which was set to [−45°, 45°], and explored the relationship between the registration results and the initial translation distance by sequentially changing the initial translation distance from [−0.1, 0.1] to [−0.5, 0.5]. The experimental results are shown in Figure 6 below:

From the above Figure 6, it can be seen that all of the algorithms are insensitive to the change in the initial translation distance, except for the ICP algorithm, where the error increases significantly as the change in the initial translation distance increases. For the rotation error, our method, DCP, and the PCRNet algorithm do not increase with the increase of the initial translation distance and they almost remain at a stable value, while for the translation error, they increase slightly with the increase in the initial translation distance. Therefore, our method is extremely robust to different initial rotations and initial translation distances without multiple iterations, and it outperforms the multiple iteration algorithms ICP and PCRNet.

### 4.3. The Effect of Different Overlapping Area Regions on the Results

To further verify the effectiveness and robustness of our algorithm with fewer overlapping regions, we verified the interval of an overlapping region from 0.5 to 0.8.

In Table 5, when the overlapping area is below 0.6, our algorithm has many cases of registration failure, and the error is increased by 100.1% compared with that when the overlap is 0.5. When the overlapping area is above 0.6, the error is in an acceptable range. Therefore, our algorithm is more suitable for an interval range with an overlapping region above 0.6. In this range, our algorithm can perform registration more efficiently.

### 4.4. Ablation Experiments

The performance improvement of our algorithm is mainly attributed to four key module designs: coarse-to-fine registration two-stage module (CFRM), multi-scale point cloud feature extraction module (MSFEM), and the attention structures modules Transformer and CBAM. To verify the effectiveness of each module, we conducted ablation experiments. The validity of the module was tested by replacing or deleting the module in each experiment while ensuring that all other experimental conditions remained unchanged. We used the rotated mean square error (MSE) of the final registration as the basis for judging validity.

In Table 6, the results of the ablation experiments show that all of these modules we designed play a positive role in the enhancement of the registration network. The multi-scale point cloud feature extraction module we designed improves the results more than 60% as compared to the basic PointNet coding module, which indicates that acquiring more local features and correlation information can substantially improve the capability of the registration network. Several other modules can further increase the accuracy of point cloud registration. It can be seen that all four modules play an integral role in enhancing the performance of the network to some extent.

## 5. Conclusions

In this paper, we investigated point cloud registration algorithms in complex situations and proposed a coarse-to-fine end-to-end point cloud registration network CCRNet. This network obtains the multi-scale features required for the registration with the help of the designed feature extraction module, then the MLPs predict the coarse registration of the quaternions and the SVD method calculates the fine registration of the rotation matrices. Finally, the two point clouds are subjected to an accurate registration operation after two rotations and translations. CCRNet solves the point cloud registration task where the registration fails or leads to incorrect matching results due to excessive initial differences, high noise, and incomplete overlap. And the experiments proved that our CCRNet method achieves better results in the point cloud registration task at the baseline and is still able to work fast and robustly with high accuracy under these complex conditions, even without any iterations.

## Figures and Tables

**Table 1:** Point cloud registration results unseen in the same categories on the ModelNet clean dataset. The best results are highlighted in bold (lower is better).

Methods	MSE(R)	RMSE(R)	MAE(R)	MSE(t)	RMSE(t)	MAE(t)	TIME(s)
ICP(P2P) [11]	10.947370	3.308681	2.517660	0.069160	0.262983	0.021960	0.031
Generalized-ICP [44]	10.600160	3.255789	2.022742	0.073520	0.271146	0.015420	0.082
PointNet-LK [8]	11.531400	3.395792	2.656400	0.082380	0.287019	0.020400	0.064
PCRNet [9]	4.629300	2.151581	1.531110	0.025940	0.161059	0.009410	0.057
DCPv1 [10]	7.624540	2.761257	1.689656	0.000205	0.014318	0.010835	**0.027**
DCPv2 [10]	2.717369	1.648444	0.979240	0.000134	0.011580	0.008505	0.035
FDCP [45]	0.000124	0.011135	0.000640	0.000001	0.003160	0.001810	12.15
Ours (CCRNet)	**2.185389**	**1.478306**	**0.900615**	**0.000125**	**0.011180**	**0.008482**	0.040

**Table 2:** Point cloud registration results unseen in the same categories on the ModelNet noisy dataset. The best results are highlighted in bold (lower is better).

Methods	MSE(R)	RMSE(R)	MAE(R)	MSE(t)	RMSE(t)	MAE(t)
ICP [11]	9.417890	3.068860	2.640210	0.088700	0.297825	0.027700
Generalized-ICP [44]	8.150900	2.854980	1.881210	0.091270	0.302109	0.023260
PCRNet [9]	4.214110	2.052830	0.944300	0.027180	0.164864	0.009410
DCPv1 [10]	7.263767	2.695138	1.691554	0.000232	0.015232	0.011358
DCPv2 [10]	2.694659	1.641542	0.918025	0.000105	0.010247	0.007796
Ours (CCRNet)	**2.251652**	**1.500551**	**0.856361**	**0.00099**	**0.009925**	**0.007579**

**Table 3:** Point cloud registration results after adding the Gaussian noise from weak to strong.

Methods	ICP		FDCP		Ours (CCRNet)	
Sigma	MAE(R)	TIME(s)	MAE(R)	TIME(s)	MAE(R)	TIME(s)
0.01	2.64021	0.02993	2.14	384	0.858245	0.03974
0.02	2.79802	0.03024	2.232	664	0.885979	0.03992
0.03	3.05914	0.03012	4.57	873	0.981902	0.03904
0.05	3.59124	0.02981	6.73	877	1.009078	0.03967
0.1	4.39743	0.03007	13.62	865	1.596119	0.04012

**Table 4:** Point cloud registration results unseen in the same categories on the incomplete overlapping dataset. The best results are highlighted in bold (lower is better).

Methods	MSE(R)	RMSE(R)	MAE(R)	MSE(t)	RMSE(t)	MAE(t)
ICP [11]	12.51042	3.536948	3.3844	0.1243	0.352562	0.03584
Generalized-ICP [44]	12.87378	3.588005	5.06372	0.12722	0.356679	0.05631
PCRNet [9]	15.7834	3.972833	4.45132	0.00291	0.053944	0.04642
DCPv1 [10]	25.78643	5.078034	3.578882	0.005004	0.070739	0.058286
DCPv2 [10]	16.25156	4.031323	2.709049	0.002836	0.053254	0.043401
Ours (CCRNet)	**10.30937**	**3.21082**	**2.202929**	**0.002258**	**0.047518**	**0.038064**

**Table 5:** Registration results for different overlapping regions from 0.5 to 0.8.

Overlap	0.5	0.6	0.7	0.8
MAE(R)	7.29	3.63	2.41	2.20
MAE(t)	0.084	0.051	0.042	0.038

**Table 6:** Ablation experiments on dataset ModelNet40. Enhanced percent is the influence of the module on the overall promotion.

	CCRNet w/o CFRM + SVD	CCRNet w/o MSFEM + PointNet	CCRNet w/o Transformer	CCRNet w/o CBAM
MSE(R)	2.63187	3.54291	2.93541	2.4345
Enhance Percent	20.43%	62.11%	34.31%	11.39%

## Data Availability

ModelNet40 dataset (https://modelnet.cs.princeton.edu/, accessed on 5 December 2023).
